# Padovan numbers which are palindromic concatenations of two distinct repdigits

**DOI:** 10.1007/s13398-021-01047-x

**Published:** 2021-04-24

**Authors:** Taboka P. Chalebgwa, Mahadi Ddamulira

**Affiliations:** 1 Department of Mathematics and Statistics, McMaster University, Hamilton, ON L8S 4K1 Canada; 2 Institute of Analysis and Number Theory, Graz University of Technology, Kopernikusgasse 24/II, 8010 Graz, Austria; 3 Max Planck Institute for Software Systems, Saarland Informatics Campus, Campus E1 5, 66123 Saarbrücken, Germany; 4 Department of Mathematics, School of Physical Sciences, College of Natural Sciences, Makerere University, PO Box 7062, Kampala, Uganda

**Keywords:** Padovan numbers, Repdigits, Linear forms in logarithms, Baker–Davenport reduction method, 11B39, 11D61, 11J86

## Abstract

In this paper we determine all Padovan numbers that are palindromic concatenations of two distinct repdigits.

## Introduction

Let $(P_n)_{n \ge 0}$ be the sequence of Padovan numbers, given by $P_{n+3} = P_{n+1} + P_{n}$, for $n \ge 0$, where $P_0 = 0$ and $P_1 = P_2 = 1$. The first few terms of this sequence are

$$\begin{aligned} (P_n)_{n\ge 3} = \{1, 2, 2, 3, 4, 5, 7, 9, 12, 16, 21, 28, 37, 49, 65, 86, 114, 151, 200, \ldots \}. \end{aligned}$$

A *repdigit* (in base 10) is a positive integer *N* that has only one distinct digit. That is, the decimal expansion of *N* takes the form

$$\begin{aligned} N = \overline{\underbrace{d\cdots d}_{\ell ~\text {times}}} = d \left( \frac{10^\ell - 1}{9} \right) , \end{aligned}$$

for some positive integers *d* and $\ell$ with $0 \le d \le 9$ and $\ell \ge 1$. This paper is a contribution to the rather well studied topic of Diophantine properties of certain linear recurrence sequences. More specifically, our paper is a variation on the theme focusing on representations of terms of a recurrent sequence as concatenations of members of another (possibly the same) sequence. For a general study of the results underpinning this topic, we direct the reader to the paper [2] by Luca and Banks, wherein (as a consequence of their level of generality) some ineffective (but finiteness) results were obtained on the number of terms of certain binary recurrent sequences whose digital representation consists of members of the same sequence.

In Ref. [1], the authors considered Fibonnaci numbers which are concatenations of two repdigits (in base 10) and showed that the largest such number is $F_{14} = 377$. Recently, diophantine equations involving Padovan numbers and repdigits have also been studied. In Ref. [12], the authors found all repdigits that can be written as a sum of two Padovan numbers. This result was later extended to repdigits that are a sum of three Padovan numbers by the second author in Ref. [8]. In Ref. [9], in the direction similar to the one in Ref. [1], the second author considered all Padovan numbers that can be written as a concatenation of two distinct repdigits and showed that the largest such number is $P_{21} = 200$. More specifically, it was shown that if $P_n$ is a solution of the Diophantine equation $P_n = \overline{\underbrace{d_1\cdots d_1}_{\ell ~\text {times}} \underbrace{d_2\cdots d_2}_{m~\text {times}}}$, times, then

$$\begin{aligned} P_n \in \{12,16,21,28,37,49,65,86,114,200 \}. \end{aligned}$$

Other related interesting results in this research direction include: the result of Bednařík and Trojovská [3], the result of Boussayoud, et al. [4], the result of Bravo and Luca [5], the result of the second author [7], the result of Erduvan and Keskin [11], the result of Rayaguru and Panda [16], the results of Trojovský [17, 18], and the result of Qu and Zeng [15]. A natural continuation of the result in Ref. [9] would be a characterization of *palindromic* Padovan numbers. As a first step in this direction, we (for the time being) consider the (more restrictive) Diophantine equation

1 $$\begin{aligned} P_n= & {} \overline{\underbrace{d_1\cdots d_1}_{\ell ~\text {times}}\underbrace{d_2\cdots d_2}_{m~\text {times}}\underbrace{d_1\cdots d_1}_{\ell ~\text {times}}},\nonumber \\&\quad \text {where} \quad d_1,d_2 \in \{0,1,2, \ldots , 9 \}, \quad d_1 > 0. \end{aligned}$$

Our result is the following.

### Theorem 1

The only Padovan numbers which are palindromic concatenations of two distinct repdigits are

$$\begin{aligned} P_n \in \{ 151, 616 \}. \end{aligned}$$

## Preliminary results

In this section we collect some facts about Padovan numbers and other preliminary lemmas that are crucial to our main argument. This preamble to the main result is similar to the one in Ref. [9] and is included here for the sake of completeness.

### Some properties of the Padovan numbers

Recall that the characteristic equation of the Padovan sequence is given by $\phi (x) := x^3 - x - 1=0$, with roots $\alpha$, $\beta$, and $\gamma = {\overline{\beta }}$ given by:

$$\begin{aligned} \alpha = \frac{r_1 + r_2}{6} \quad \text {and} \quad \beta = \frac{-(r_1 + r_2) + i \sqrt{3}(r_1 - r_2)}{12}, \end{aligned}$$

where

$$\begin{aligned} r_1 = \root 3 \of {108 + 12 \sqrt{69}} \quad \text {and} \quad r_2 = \root 3 \of {108 - 12 \sqrt{69}}. \end{aligned}$$

For all $n \ge 0$, Binet’s formula for the Padovan sequence tells us that the *n*th Padovan number is given by

2 $$\begin{aligned} P_n = a\alpha ^n + b \beta ^n + c\gamma ^n, \end{aligned}$$

where

$$\begin{aligned} a = \frac{\alpha + 1}{(\alpha - \beta )(\alpha - \gamma )}, \quad b = \frac{\beta + 1}{(\beta - \alpha )(\beta - \gamma )}, \quad \text {and} \quad c = \frac{\gamma +1}{(\gamma - \alpha )(\gamma - \beta )}={\bar{b}}. \end{aligned}$$

The minimal polynomial of *a* over ${\mathbb {Z}}$ is given by

$$\begin{aligned} 23x^3 - 5x - 1, \end{aligned}$$

and its zeros are *a*, *b*, *c* as given above. One can check that $|a|, |b|, |c| < 1$. Numerically, we have the following estimates for the quantities $\{\alpha , \beta , \gamma , a,b,c \}$:

3 $$\begin{aligned} \begin{aligned} 1.32<&\alpha< 1.33, \\ 0.86< |\beta | =&|\gamma | = \alpha ^{-\frac{1}{2}}< 0.87, \\ 0.54<&a< 0.55, \\ 0.28< |b|&= |c| < 0.29. \end{aligned} \end{aligned}$$

It follows that the contribution to the right hand side of Eq. (2) due to the complex conjugate roots $\beta$ and $\gamma$ is small. More specifically, let

4 $$\begin{aligned} e(n) := P_n - a \alpha ^n = b \beta ^n + c\gamma ^n. \quad \text {Then,} \quad |e(n)| < \frac{1}{\alpha ^{n/2}} \quad \text {for all} \quad n \ge 1. \end{aligned}$$

The last inequality in (4) follows from the fact that $|\beta |=|\gamma |=\alpha ^{-\frac{1}{2}}$ and $|b|=|c|< 0.29$ (by (3)). That is, for any $n\ge 1$,

$$\begin{aligned} |e(n)|=\left| b\beta ^{n}+c\gamma ^{n}\right| \le |b||\beta |^{n}+ |c||\gamma |^{n}= |b|\alpha ^{-\frac{n}{2}}+ |c| \alpha ^{-\frac{n}{2}}< 2\cdot 0.29 \cdot \alpha ^{-\frac{n}{2}} < \dfrac{1}{\alpha ^{n/2}}. \end{aligned}$$

Furthermore, the following estimate also holds:

#### Lemma 1

Let $n \ge 1$ be a positive integer. Then

$$\begin{aligned} \alpha ^{n-3} \le P_n \le \alpha ^{n-1}. \end{aligned}$$

Lemma 1 follows from a simple inductive argument.

Let ${\mathbb {K}} := {\mathbb {Q}}(\alpha , \beta )$ be the splitting field of the polynomial $\phi$ over ${\mathbb {Q}}$. Then $[{\mathbb {K}}: {\mathbb {Q}}] = 6$ and $[{\mathbb {Q}}(\alpha ): {\mathbb {Q}}] = 3$. We note that the Galois group of ${\mathbb {K}}/{\mathbb {Q}}$ is given by

$$\begin{aligned} {\mathcal {G}} := \text {Gal}({\mathbb {K}}/{\mathbb {Q}}) \cong \{ (1), (\alpha \beta ), (\alpha \gamma ), (\beta \gamma ), (\alpha \beta \gamma ) \} \cong S_3. \end{aligned}$$

Therefore, we identify the automorphisms of ${\mathcal {G}}$ with the permutation group of the zeroes of $\phi$. We shall find particular use for the permutation $(\alpha \beta )$, corresponding to the automorphism $\sigma : \alpha \mapsto \beta , \beta \mapsto \alpha , \gamma \mapsto \gamma$.

### Linear forms in logarithms

Like many proofs of similar results, the crucial steps in our argument involve obtaining certain bounds on linear forms in (nonzero) logarithms. The upper bounds usually follow easily from a manipulation of the associated Binet’s formula for the sequence in question. For the lower bounds, we need the celebrated Baker’s theorem on linear forms in logarithms. Before stating the result, we need the definition of the (logarithmic) Weil height of an algebraic number.

Let $\eta$ be an algebraic number of degree *d* with minimal polynomial

$$\begin{aligned} P(x) = a_0 \prod _{j = 1}^d (x - \alpha _j), \end{aligned}$$

where the leading coefficient $a_0$ is positive and the $\alpha _j$’s are the conjugates of $\alpha$. The logarithmic height of $\eta$ is given by

$$\begin{aligned} h(\eta ) := \frac{1}{d} \left( \log a_0 + \sum _{j = 1}^d \log \left( \max \{|\alpha _j|, 1 \} \right) \right) . \end{aligned}$$

Note that, if $\eta = \frac{p}{q} \in {\mathbb {Q}}$ is a reduced rational number with $q > 0$, then the above definition reduces to $h(\eta ) = \log \max \{ |p|,q \}$. We list some well known properties of the height function below, which we shall subsequently use without reference:

$$\begin{aligned} h(\eta _1 \pm \eta _2)&\le h(\eta _1) + h(\eta _2) + \log 2, \\ h(\eta _1 \eta _2 ^{\pm 1})&\le h(\eta _1) + h(\eta _2), \\ h(\eta ^s)&= |s| h(\eta ), \quad (s \in {\mathbb {Z}}). \end{aligned}$$

We quote the version of Baker’s theorem proved by Bugeaud, Mignotte, and Siksek ([6], Theorem 9.4).

#### Theorem 2

Let $\eta _1, \ldots , \eta _t$ be positive real algebraic numbers in a real algebraic number field ${\mathbb {K}} \subset {\mathbb {R}}$ of degree *D*. Let $b_1, \ldots , b_t$ be nonzero integers such that

$$\begin{aligned} \Gamma := \eta _1 ^{b_1} \ldots \eta _t ^{b_t} - 1 \ne 0. \end{aligned}$$

Then

$$\begin{aligned} \log | \Gamma | > - 1.4 \cdot 30^{t+3}\cdot t^{4.5} \cdot D^2 (1 + \log D)(1 + \log B)A_1 \ldots A_t, \end{aligned}$$

where

$$\begin{aligned} B \ge \max \{|b_1|, \ldots , |b_t| \} \end{aligned}$$

and

$$\begin{aligned} A_j \ge \max \{ D h(\eta _j), |\log \eta _j|, 0.16 \}, \quad \text {for all} \quad j = 1, \ldots ,t. \end{aligned}$$

### Baker–Davenport reduction

The bounds on the variables obtained via Baker’s theorem are usually too large for any computational purposes. In order to get further refinements, we use the Baker–Davenport reduction procedure. The variant we apply here is the one due to Dujella and Pethö ([10], Lemma 5a). For a real number *r*, we denote by $\parallel r \parallel$ the quantity $\min \{|r - n| : n \in {\mathbb {Z}} \}$, which is the distance from *r* to the nearest integer.

#### Lemma 2

Let $\kappa \ne 0$, and $A, B, \mu$ be real numbers with $A > 0$ and $B > 1$. Let $M > 1$ be a positive integer and suppose that $\frac{p}{q}$ is a convergent of the continued fraction expansion of $\kappa$ with $q > 6M$. Let

$$\begin{aligned} \varepsilon := \parallel \mu q \parallel - M \parallel \kappa q \parallel . \end{aligned}$$

If $\varepsilon > 0$, then there is no solution of the inequality

$$\begin{aligned} 0< |m \kappa - n + \mu | < AB^{-k} \end{aligned}$$

in positive integers *m*, *n*, *k* with

$$\begin{aligned} \frac{\log (Aq/\varepsilon )}{\log B} \le k \quad \text {and} \quad m \le M. \end{aligned}$$

We will also need the following lemma by Gúzman Sánchez and Luca ([13], Lemma 7):

#### Lemma 3

Let $r \ge 1$ and $H > 0$ be such that $H > (4r^2)^r$ and $H > L/(\log L)^r$. Then

$$\begin{aligned} L < 2^r H (\log H)^r. \end{aligned}$$

## Proof of the main result

### The low range

With the help of a simple computer program in Mathematica, we checked all the solutions to the Diophantine equation (1) in the ranges $d_1\ne d_2 \in \{0,1,2, \ldots , 9\},~ d_1>0$ and $1\le \ell ,m \le n\le 1000$. We found only the solutions stated in Theorem 1. Here onwards, we assume that $n>1000$.

### The initial bound on *n*

We note that (1) can be rewritten as

5 $$\begin{aligned} P_n&= \overline{\underbrace{d_1\cdots d_1}_{\ell ~\text {times}}\underbrace{d_2\cdots d_2}_{m~\text {times}}\underbrace{d_1\cdots d_1}_{\ell ~\text {times}}} \nonumber \\&= \overline{\underbrace{d_1\cdots d_1}_{\ell ~\text {times}} \cdot 10^{\ell + m} + \underbrace{d_2\cdots d_2}_{m~\text {times}} \cdot 10^\ell + \underbrace{d_1\cdots d_1}_{\ell ~\text {times}}} \nonumber \\&= \frac{1}{9} \left( d_1 \cdot 10^{2\ell + m} - (d_1 - d_2) \cdot 10^{\ell +m} + (d_1-d_2) \cdot 10^\ell - d_1 \right) . \end{aligned}$$

The next lemma relates the sizes of *n* and $2\ell + m$.

#### Lemma 4

All solutions of (5) satisfy

$$\begin{aligned} (2\ell + m) \log 10 - 3< n \log \alpha < (2\ell + m) \log 10 + 1. \end{aligned}$$

#### Proof

Recall that $\alpha ^{n-3} \le P_n \le \alpha ^{n-1}$. We note that

$$\begin{aligned} \alpha ^{n-3} \le P_n < 10^{2\ell + m}. \end{aligned}$$

Taking the logarithm on both sides, we get

$$\begin{aligned} n \log \alpha < (2\ell + m) \log 10 + 3 \log \alpha . \end{aligned}$$

Hence, $n \log \alpha < (2\ell + m) \log 10 + 1$. The lower bound follows via the same technique, upon noting that $10^{2\ell + m -1} < P_n \le \alpha ^{n-1}$. $\square$

We proceed to examine (5) in three different steps as follows.

**Step 1.** From Eqs. (2) and (5), we have that

$$\begin{aligned} 9(a \alpha ^n + b \beta ^n + c \gamma ^n )= d_1 \cdot 10^{2\ell + m} - (d_1-d_2)\cdot 10^{\ell +m} + (d_1-d_2)\cdot 10^\ell - d_1. \end{aligned}$$

Hence,

$$\begin{aligned} 9 a\alpha ^n - d_1 \cdot 10^{2\ell + m} = -9e(n) - (d_1-d_2)\cdot 10^{\ell +m} + (d_1-d_2)\cdot 10^\ell - d_1. \end{aligned}$$

We thus have that

$$\begin{aligned} |9 a\alpha ^n - d_1 \cdot 10^{2\ell + m}|&= |-9e(n) - (d_1-d_2)\cdot 10^{\ell +m} + (d_1-d_2)\cdot 10^\ell - d_1| \\&\le 9 \alpha ^{-n/2} + 27 \cdot 10^{\ell +m} \\&< 28 \cdot 10^{\ell +m}, \end{aligned}$$

where we used the fact that $n > 1000$. Dividing both sides by $d_1 \cdot 10^{2\ell + m}$, we get

6 $$\begin{aligned} \left| \left( \frac{9a}{d_1} \right) \cdot \alpha ^n \cdot 10^{-2\ell -m} - 1 \right| < \frac{28 \cdot 10^{m+\ell }}{d_1 \cdot 10^{2\ell +m}} \le \frac{28}{10^\ell }. \end{aligned}$$

We put

$$\begin{aligned} \Gamma _1 := \left( \frac{9a}{d_1} \right) \cdot \alpha ^n \cdot 10^{-2\ell -m} - 1 . \end{aligned}$$

We shall compare this upper bound on $|\Gamma _1|$ with the lower bound we deduce from Theorem 2. Note that $\Gamma _1 \ne 0$, since this would imply that $a \alpha ^n = \frac{d_1\cdot 10^{2\ell + m}}{9}$. If this is the case, then applying the automorphism $\sigma$ on both sides of the preceeding equation and taking absolute values, we have that

$$\begin{aligned} \left| \frac{d_1\cdot 10^{2\ell + m}}{9} \right| = |\sigma (a \alpha ^n)| = |b \beta ^n| < 1, \end{aligned}$$

which is false. We thus have that $\Gamma _1 \ne 0$.

With a view towards applying Theorem 2, we define the following parameters:

$$\begin{aligned} \eta _1 := \frac{9a}{d_1}, \ \eta _2 := \alpha , \ \eta _3 := 10, \ b_1 := 1, \ b_2 := n, \ b_3 := -2\ell - m, \ t: = 3. \end{aligned}$$

Since $10^{2\ell +m -1} < P_n \le \alpha ^{n-1}$, we have that $2\ell + m < n$. Thus we take $B = n$. We note that ${\mathbb {K}} = {\mathbb {Q}}(\eta _1, \eta _2, \eta _3) = {\mathbb {Q}}(\alpha )$, since $a = \alpha (\alpha + 1)/(2 \alpha +3)$. Hence $D = [{\mathbb {K}}: {\mathbb {Q}}] = 3$.

We note that

$$\begin{aligned} h(\eta _1) = h \left( \frac{9a}{d_1} \right) \le 2h(9) + h(a) \le 2\log 9 + \frac{1}{3}\log 23 < 5.44. \end{aligned}$$

We also have that $h(\eta _2) = h(\alpha ) = \frac{1}{3}\log \alpha$ and $h(\eta _3) = \log 10$. Hence, we let

$$\begin{aligned} A_1 := 16.32, \ A_2 := \log \alpha , \ A_3 := 3 \log 10. \end{aligned}$$

Thus, we deduce via Theorem 2 that

$$\begin{aligned} \log |\Gamma |&> -1.4 \cdot 30^6 3^{4.5} 3^2 (1 + \log 3)(1 + \log n)(16.32)(\log \alpha )(3 \log 10) \\ {}&> -1.45\cdot 10^{30}(1 + \log n). \end{aligned}$$

Comparing the last inequality obtained above with (6), we get

$$\begin{aligned} \ell \log 10 - \log 28 < 1.45 \cdot 10^{30}(1 + \log n), \end{aligned}$$

and therefore,

7 $$\begin{aligned} \ell \log 10 < 1.46 \cdot 10^{30}(1 + \log n). \end{aligned}$$

**Step 2.** We rewrite Eq. (5) as

$$\begin{aligned} 9 a\alpha ^n - d_1 \cdot 10^{2\ell + m} + (d_1-d_2)\cdot 10^{m+\ell } = -9e(n) + (d_1-d_2)\cdot 10^\ell - d_1. \end{aligned}$$

That is,

$$\begin{aligned} 9 a\alpha ^n - (d_1 \cdot 10^\ell - (d_1-d_2))\cdot 10^{m+\ell } = -9e(n) + (d_1-d_2)\cdot 10^\ell - d_1. \end{aligned}$$

Hence,

$$\begin{aligned} |9 a\alpha ^n - (d_1 \cdot 10^\ell - (d_1-d_2))\cdot 10^{m+\ell }|&= |-9e(n) + (d_1-d_2)\cdot 10^\ell - d_1|\\&\le \frac{9}{\alpha ^{n/2}} + 18 \cdot 10^\ell < 19 \cdot 10^\ell . \end{aligned}$$

Dividing throughout by $(d_1 \cdot 10^\ell - (d_1-d_2))\cdot 10^{m+\ell }$, we have that

8 $$\begin{aligned} \left| \left( \frac{9a}{d_1 \cdot 10^\ell - (d_1-d_2)} \right) \cdot \alpha ^n \cdot 10^{-\ell - m} -1 \right|< \frac{19 \cdot 10^\ell }{(d_1 \cdot 10^\ell - (d_1-d_2))\cdot 10^{m+\ell }} < \frac{19}{10^m}. \end{aligned}$$

We put

$$\begin{aligned} \Gamma _2: = \left( \frac{9a}{d_1 \cdot 10^\ell - (d_1-d_2)} \right) \cdot \alpha ^n \cdot 10^{-\ell -m} - 1. \end{aligned}$$

As before, we have that $\Gamma _2 \ne 0$ because this would imply that

$$\begin{aligned} a \alpha ^n = 10^{m + \ell } \left( \frac{d_1 \cdot 10^\ell - (d_1-d_2)}{9} \right) , \end{aligned}$$

which in turn implies that

$$\begin{aligned} \left| 10^{m + \ell } \left( \frac{d_1 \cdot 10^\ell - (d_1-d_2)}{9} \right) \right| = |\sigma ( a \alpha ^n)| = |b \beta ^n | < 1, \end{aligned}$$

which is false. In preparation towards applying Theorem 2, we define the following parameters:

$$\begin{aligned} \eta _1: = \frac{9a}{d_1 \cdot 10^\ell - (d_1-d_2)} , \ \eta _2: = \alpha , \ \eta _3: = 10, \ b_1: = 1, \ b_2 := n, \ b_3 := -\ell - m, \ t := 3. \end{aligned}$$

In order to determine what $A_1$ will be, we need to find the find the maximum of the quantities $h(\eta _1)$ and $|\log \eta _1|$.

We note that

$$\begin{aligned} h(\eta _1)&= h \left( \frac{9a}{d_1 \cdot 10^\ell - (d_1-d_2)} \right) \\&\le h(9) + h(a) + \ell h(10) +h(d_1) + h(d_1-d_2) + \log 2\\&\le 3 \log 9 + h(a) + \ell \log 10\\&< 3\log 9 + \frac{1}{3} \log 23 + 1.46\cdot 10^{30} (1+ \log n)\\&< 1.48\cdot 10^{30}(1 + \log n), \end{aligned}$$

where in the second last inequality above, we used (7). On the other hand, we also have that

$$\begin{aligned} |\log \eta _1|&= \left| \log \left( \frac{9a}{d_1 \cdot 10^\ell - (d_1-d_2)} \right) \right| \\&\le \log a + \log 9 + |\log (d_1 \cdot 10^\ell - (d_1-d_2))|\\&\le \log a + \log 9 + \log (d_1 \cdot 10^\ell ) + \left| \log \left( 1 - \frac{d_1 - d_2}{d_1 \cdot 10^\ell } \right) \right| \\&\le \ell \log 10 + \log d_1 + \log 9 + \log (0.55) +\left| \frac{|d_1 - d_2|}{d_1 \cdot 10^\ell } + \frac{1}{2} \left( \frac{|d_1 - d_2|}{d_1 \cdot 10^\ell } \right) ^2 + \cdots \right| \\&\le \ell \log 10 + 3 \log 9 + \frac{1}{10^\ell } + \frac{1}{2 \cdot 10^{2\ell }} + \cdots \\&\le 1.46 \cdot 10^{30}(1 + \log n) + 3 \log 9 + \frac{1}{10^\ell - 1} \\&< 1.48 \cdot 10^{30}(1 + \log n), \end{aligned}$$

where in the second last inequality, we used Eq. (7). We note that $D h(\eta _1) > |\log \eta _1|$.

We thus let $A_1 := 4.44 \cdot 10^{30} (1 + \log n)$. We take $A_2: = \log \alpha$ and $A_3 := 3 \log 10$, as defined in Step 1. Similarly, we take $B := n$.

Theorem 2 then tells us that

$$\begin{aligned} \log |\Gamma _2|&> -1.4 \cdot 30^6 \cdot 3^{4.5} \cdot 3^2 \cdot (1 + \log 3)(1 + \log n)(\log \alpha )(3 \log 10)A_1\\&> -2 \cdot 10^{13}(1 + \log n)A_1 ~~ > -8.88 \cdot 10^{43}(1 + \log n)^2. \end{aligned}$$

Comparing the last inequality with (8), we have that

9 $$\begin{aligned} m \log 10&< 8.88 \cdot 10^{43}(1 + \log n)^2 + \log 19 \nonumber \\&< 9 \cdot 10^{43}(1+\log n)^2. \end{aligned}$$

**Step 3.** We rewrite Eq. (5) as

$$\begin{aligned} 9 a\alpha ^n - d_1 \cdot 10^{2\ell + m} + (d_1-d_2)\cdot 10^{m+\ell } - (d_1-d_2)\cdot 10^\ell = -9e(n) - d_1. \end{aligned}$$

Therefore,

$$\begin{aligned} \left| 9a\alpha ^n - (d_1 \cdot 10^{\ell +m} + (d_1-d_2)\cdot 10^m - (d_1-d_2))\cdot 10^\ell \right|&= |-9e(n) - d_1|\\&\le \frac{9}{\alpha ^{n/2}} + 9 ~~ < 10. \end{aligned}$$

Consequently,

10 $$\begin{aligned} \left| \left( \frac{1}{9a} \right) (d_1 \cdot 10^{\ell +m} + (d_1-d_2)\cdot 10^m - (d_1-d_2))\cdot \alpha ^{-n} \cdot 10^\ell - 1 \right|< \frac{10}{9 a\alpha ^n} < \frac{3}{\alpha ^n}.\nonumber \\ \end{aligned}$$

Let

$$\begin{aligned} \Gamma _3 := \left[ \left( \frac{1}{9a} \right) (d_1 \cdot 10^{\ell +m} + (d_1-d_2)\cdot 10^m - (d_1-d_2)) \right] \cdot \alpha ^{-n} \cdot 10^\ell - 1. \end{aligned}$$

As before, we have that $\Gamma _3 \ne 0$ since we would have that

$$\begin{aligned} a \alpha ^n = \frac{1}{9} \left( d_1 \cdot 10^{\ell +m} + (d_1-d_2)\cdot 10^m - (d_1-d_2)\right) \cdot 10^\ell . \end{aligned}$$

Applying the automorphism $\sigma$ from the Galois group ${\mathcal {G}}$ on both sides of the above equation and then taking absolute values, we have that

$$\begin{aligned} \left| \frac{1}{9} \left( d_1 \cdot 10^{\ell +m} + (d_1-d_2)\cdot 10^m - (d_1-d_2)\right) \cdot 10^\ell \right| = |\sigma \left( a \alpha ^n\right) | = |b \beta ^n| < 1, \end{aligned}$$

which is false. We would now like to apply Theorem 2 to $\Gamma _3$. To this end, we let:

$$\begin{aligned} \eta _1&:= \left[ \left( \frac{1}{9a} \right) \left( d_1 \cdot 10^{\ell +m} + (d_1-d_2)\cdot 10^m - (d_1-d_2)\right) \right] , \ \eta _2 := \alpha , \ \eta _3 := 10, \\&\qquad \qquad b_1 := 1, \ b_2: = -n, \ b_3: = \ell , \ t: = 3. \end{aligned}$$

As in the previous cases, we can take $B: = n$ and $D: = 3$. We note that

$$\begin{aligned} h(\eta _1)&\le h(9) + h(a) + h(d_1) + (\ell +m)h(10) + h(d_1 - d_2) \\&\quad + m h(10) + h(d_1 - d_2) + 3 \log 2\\&\le 5 \log 9 + \frac{\log 23}{3} + (\ell + m) \log 10 + m \log 10 \\&\le 6 \log 9 + (\ell +m)\log 10 + m \log 10. \end{aligned}$$

Using Eqs. (7) and (9), we have that

11 $$\begin{aligned} (\ell +m)\log 10&< 1.46\cdot 10^{30} (1 + \log n) + 9 \cdot 10^{43}(1 + \log n)^2 \nonumber \\&< 10 \cdot 10^{43}(1 + \log n)^2. \end{aligned}$$

Thus, we deduce that

$$\begin{aligned} h(\eta _1) < 20 \cdot 10^{43}(1 + \log n)^2. \end{aligned}$$

We now find an upper bound for $|\log \eta _1|$. We have that

$$\begin{aligned} |\log \eta _1|&= \left| \log \left( \left( \frac{1}{9a} \right) \left( d_1 \cdot 10^{\ell +m} + (d_1-d_2)\cdot 10^m - (d_1-d_2)\right) \right) \right| \\&\le \log 9 + \log a + |\log (d_1 \cdot 10^{\ell +m} + (d_1-d_2)\cdot 10^m - (d_1-d_2))|\\&\le 2 \log 9 + \log (d_1 \cdot 10^{\ell + m}) + \left| \log \left( 1 - \frac{(d_1 - d_2)(10^m - 1)}{d_1 \cdot 10^{\ell +m}} \right) \right| \\&\le 3 \log 9 + (\ell +m) \log 10 + \left| \log \left( 1 - \frac{(d_1 - d_2)(10^m - 1)}{d_1 \cdot 10^{\ell +m}} \right) \right| \\&\le 3 \log 9 + (\ell +m) \log 10\\&\qquad +\left| \frac{|(d_1 - d_2)(10^m - 1)|}{d_1 \cdot 10^{\ell + m}} + \frac{1}{2} \left( \frac{|(d_1 - d_2)(10^m - 1)|}{d_1 \cdot 10^{\ell + m}} \right) ^2 + \cdots \right| \\&\le 3 \log 9 + (\ell +m) \log 10 + \frac{1}{10^\ell } + \frac{1}{2 \cdot 10^{2\ell }} + \cdots \\&< 3 \log 9 + (\ell +m)\log 10 + \frac{1}{10^\ell - 1}\\&< 1.1 \cdot 10^{44}(1 + \log n)^2, \end{aligned}$$

where in the last inequality above, we used the bound from (11). We note that $D \cdot h(\eta _1) > |\log \eta _1|$. We thus let $A_1: = 6 \cdot 10^{44}(1 + \log n)^2$, $A_2: = \log \alpha$ and $3 \log 10$. Theorem 2 then implies that

$$\begin{aligned} \log |\Gamma _3|> -2 \cdot 10^{13} (1 + \log n)A_1> -1.2 \cdot 10^{58} (1 + \log n)^3. \end{aligned}$$

Comparing the last inequality with (10), we deduce that

$$\begin{aligned} n \log \alpha < 1.2 \cdot 10^{58} (1 + \log n)^3 + \log 3. \end{aligned}$$

It follows that

$$\begin{aligned} n < 5 \cdot 10^{58} (\log n)^3. \end{aligned}$$

With the notation of Lemma 3, we let $r: = 3$, $L := n$, and $H: = 5 \cdot 10^{58}$ and notice that this data meets the conditions of the lemma. Applying the lemma, we have that

$$\begin{aligned} n < 2^3 \cdot 5 \cdot 10^{58} (\log (5 \cdot 10^{58}))^3. \end{aligned}$$

After a simplification, we obtain the (rather loose) bound

$$\begin{aligned} n < 1.04 \cdot 10^{66}. \end{aligned}$$

Lemma 4 then implies that

$$\begin{aligned} 2\ell +m < 1.4 \cdot 10^{65}. \end{aligned}$$

The following lemma summarizes what we have proved thus far:

#### Lemma 5

All solutions to the Diophantine equation (1) satisfy

$$\begin{aligned} 2\ell + m< 1.4 \cdot 10^{65} \quad \text {and} \quad n < 1.04 \cdot 10^{66}. \end{aligned}$$

### The reduction procedure

The bounds obtained in Lemma 5 are too large to be useful computationally. Thus, we need to reduce them. To do so, we apply Lemma 2 as follows. First, we return to the inequality (6) and put

$$\begin{aligned} z_1:=(2\ell +m)\log 10 -n\log \alpha +\log \left( \frac{d_1}{9a}\right) . \end{aligned}$$

The inequality (6) can be rewritten as

$$\begin{aligned} |\Gamma _1|=\left| e^{-z_1}-1\right| < \dfrac{28}{10^{\ell }}. \end{aligned}$$

If we assume that $\ell \ge 2$, then the right–hand side of the above inequality is at most $28/100 < 1/2$. The inequality $|e^z-1|< x$ for real values of *x* and *z* implies that $z<2x$. Thus,

$$\begin{aligned} |z_1|< \dfrac{56}{10^{\ell }}. \end{aligned}$$

This implies that

$$\begin{aligned} \left| (2\ell +m)\log 10 -n\log \alpha -\log \left( \frac{9a}{d_1}\right) \right| < \dfrac{56}{10^{\ell }}. \end{aligned}$$

Dividing through the above inequality by $\log \alpha$ gives

$$\begin{aligned} \left| (2\ell +m)\frac{\log 10}{\log \alpha } - n + \left( \frac{\log (d_1/9a)}{\log \alpha }\right) \right| < \dfrac{56}{10^{\ell }\log \alpha }. \end{aligned}$$

So, we apply Lemma 2 with the quantities:

$$\begin{aligned} \kappa :=\dfrac{\log 10}{\log \alpha }, \quad \mu (d_1):=\frac{\log (d_1/9a)}{\log \alpha }, \quad 1\le d_1\le 9, \quad A:=\dfrac{56}{\log \alpha }, \quad B:=10. \end{aligned}$$

Let $\kappa =[a_0;a_1,a_2, \ldots ]=[8; 5, 3, 3, 1, 5, 1, 8, 4, 6, 1, 4, 1, 1, 1, 9, 1, 4, 4, 9, 1, 5, \ldots ]$ be the continued fraction expansion of $\kappa$. We set $M:=10^{66}$ which is the upper bound on $2\ell +m$. With the help of Mathematica, we find that the convergent

$$\begin{aligned} \dfrac{p}{q}=\dfrac{p_{141}}{q_{141}}=\dfrac{92894276795199235673676174009251522651329656614011503595729035741839}{11344567100398997770258435239827426964781308977543724537727298754290}, \end{aligned}$$

is such that $q=q_{141}>6M$. Furthermore, it gives $\varepsilon > 0.0716554$, and thus,

$$\begin{aligned} \ell \le \dfrac{\log ((56/\log \alpha )q/\varepsilon )}{\log 10}<70. \end{aligned}$$

Therefore, we have that $\ell \le 70$. The case $\ell <2$ also holds because $\ell<2<70$.

Next, for fixed $d_1\ne d_2\in \{0,1,2, \ldots , 9\}, ~d_1>0$ and $1\le \ell \le 70$, we return to the inequality (8) and put

$$\begin{aligned} z_2:=(\ell +m)\log 10 - n\log \alpha +\log \left( \frac{d_1\cdot 10^{\ell }-(d_1-d_2)}{9a}\right) . \end{aligned}$$

From the inequality (8), we have that

$$\begin{aligned} |\Gamma _2| = \left| e^{-z_2}-1\right| < \dfrac{19}{10^{m}}. \end{aligned}$$

Assume that $m\ge 2$, then the right–hand side of the above inequality is at most $19/100 < 1/2$. Thus, we have that

$$\begin{aligned} |z_2|< \dfrac{38}{10^{m}}, \end{aligned}$$

which implies that

$$\begin{aligned} \left| (\ell +m)\log 10 - n\log \alpha +\log \left( \frac{d_1\cdot 10^{\ell }-(d_1-d_2)}{9a}\right) \right| < \dfrac{38}{10^{m}}. \end{aligned}$$

Dividing through by $\log \alpha$ gives

$$\begin{aligned} \left| (\ell +m)\frac{\log 10}{\log \alpha } - n +\frac{\log \left( (d_1\cdot 10^{\ell }-(d_1-d_2))/9a\right) }{\log \alpha }\right| < \dfrac{38}{10^{m}\log \alpha }. \end{aligned}$$

Thus, we apply Lemma 2 with the quantities:

$$\begin{aligned} \quad \mu (d_1,d_2):=\frac{\log \left( (d_1\cdot 10^{\ell }-(d_1-d_2))/9a\right) }{\log \alpha }, \quad A:=\dfrac{38}{\log \alpha }, \quad B:=10. \end{aligned}$$

We take the same $$ \kappa $$ and its convergent $$ p/q =p_{141}/q_{141} $$ as before. Since $$ \ell +m < 2\ell +m $$, we set $$M :=10^{66} $$ as the upper bound on $$ \ell +m $$. With the help of a simple computer program in Mathematica, we get that $$ \varepsilon >0.0000918806 $$, and therefore,

$$\begin{aligned} m\le \dfrac{\log ((38/\log \alpha )q/\varepsilon )}{\log 10}<73. \end{aligned}$$

Thus, we have that $m\le 73$. The case $m<2$ holds as well since $m<2<73$.

Lastly, for fixed $d_1\ne d_2\in \{0,1,2, \ldots , 9\}, ~d_1>0$, $1\le \ell \le 69$ and $1\le m\le 73$, we return to the inequality (10) and put

$$\begin{aligned} z_3:=\ell \log 10 -n\log \alpha +\log \left( \dfrac{d_1\cdot 10^{\ell +m}+(d_1-d_2)\cdot 10^{m}-(d_1-d_2)}{9a}\right) . \end{aligned}$$

From the inequality (10), we have that

$$\begin{aligned} |\Gamma _3|=\left| e^{z_3}-1\right| < \frac{3}{\alpha ^{n}}. \end{aligned}$$

Since $n>1000$, the right–hand side of the above inequality is less than 1/2. Thus, the above inequality implies that

$$\begin{aligned} |z_3|< \frac{6}{\alpha ^{n}}, \end{aligned}$$

which leads to

$$\begin{aligned} \left| \ell \log 10 -n\log \alpha +\log \left( \dfrac{d_1\cdot 10^{\ell +m}+(d_1-d_2)\cdot 10^{m}-(d_1-d_2)}{9a}\right) \right| < \frac{6}{\alpha ^{n}}. \end{aligned}$$

Dividing through by $\log \alpha$ gives,

$$\begin{aligned} \left| \ell \frac{\log 10}{\log \alpha } -n +\dfrac{\log \left( (d_1\cdot 10^{\ell +m}+(d_1-d_2)\cdot 10^{m}-(d_1-d_2))/9a\right) }{\log \alpha }\right| < \frac{6}{\alpha ^{n}\log \alpha }. \end{aligned}$$

Again, we apply Lemma 2 with the quantities:

$$\begin{aligned} \quad \mu (d_1, d_2):=\dfrac{\log \left( (d_1\cdot 10^{\ell +m}+(d_1-d_2)\cdot 10^{m}-(d_1-d_2))/9a\right) }{\log \alpha }, \quad A:=\dfrac{6}{\log \alpha }, \quad B:=\alpha . \end{aligned}$$

We take the same $\kappa$ and its convergent $p/q=p_{141}/q_{141}$ as before. Since $\ell <2\ell +m$, we choose $M:=10^{66}$ as the upper bound for $\ell$. With the help of a simple computer program in Mathematica, we get that $\varepsilon > 0.00000594012$, and thus,

$$\begin{aligned} n\le \dfrac{\log ((6/\log \alpha )q/\varepsilon )}{\log \alpha }< 602. \end{aligned}$$

Thus, we have shown that $n\le 602$, contradicting our assumption that $n> 1000$. Therefore, Theorem 1 holds. $\square$
